# Prospects of photonic computing for artificial intelligence

**DOI:** 10.1093/nsr/nwag089

**Published:** 2026-02-11

**Authors:** Hailong Zhou, Jianji Dong

**Affiliations:** Wuhan National Laboratory for Optoelectronics, School of Optical and Electronic Information, Huazhong University of Science and Technology, China; Wuhan National Laboratory for Optoelectronics, School of Optical and Electronic Information, Huazhong University of Science and Technology, China

## Abstract

Photonic computing emerges as a disruptive paradigm for artificial intelligence, presenting a strategic choice between scaling linear optical engines and pursuing deep, end-to-end optical neural networks.

The increasing demand for computational power, driven by the exponential growth of artificial intelligence and machine learning, is pushing conventional electronic processors based on von Neumann architecture to their physical limits—the so-called ‘Moore’s Law is dead’ predicament. Optical computing has emerged as a disruptive paradigm, poised to overcome the challenge. By processing data in the form of photons instead of electrons, it leverages the fundamental properties of light—high bandwidth, low latency and inherent parallelism—to perform computations, particularly matrix-vector multiplications (MVMs), at speeds and efficiencies far exceeding their electronic counterparts [1]. This advantage has ignited a race to build commercially viable optical computers, leading the community to a strategic crossroads: should we prioritize scaling up the size of linear optical operators or focus on integrating nonlinearity to build deeper end-to-end optical networks?

The first path, scaling up linear optical engines, is a practical approach that has gained significant commercial traction. This strategy focuses on what optics does best: performing large-scale MVMs with remarkable speed and energy efficiency, as shown in Fig. 1a. Companies such as Lightmatter and Lightelligence have pioneered this area, developing photonic processors that can be integrated into existing electronic computing frameworks as powerful coprocessors or accelerators [2]. These systems typically use Mach–Zehnder Interferometer (MZI) meshes or micro-ring resonator (MRR) weight banks to represent a matrix, upon which input data, encoded onto the intensity or phase of coherent light, is multiplied in a single shot. Current linear acceleration architectures have achieved a throughput of 11 TOPS [3]. Leveraging multiplexing across dimensions such as wavelength, mode and polarization offers a promising pathway to further enhance computational density. The primary application for these processors is accelerating the linear-heavy workloads in deep-learning inference, such as convolutional and fully connected layers.

**Figure 1.**
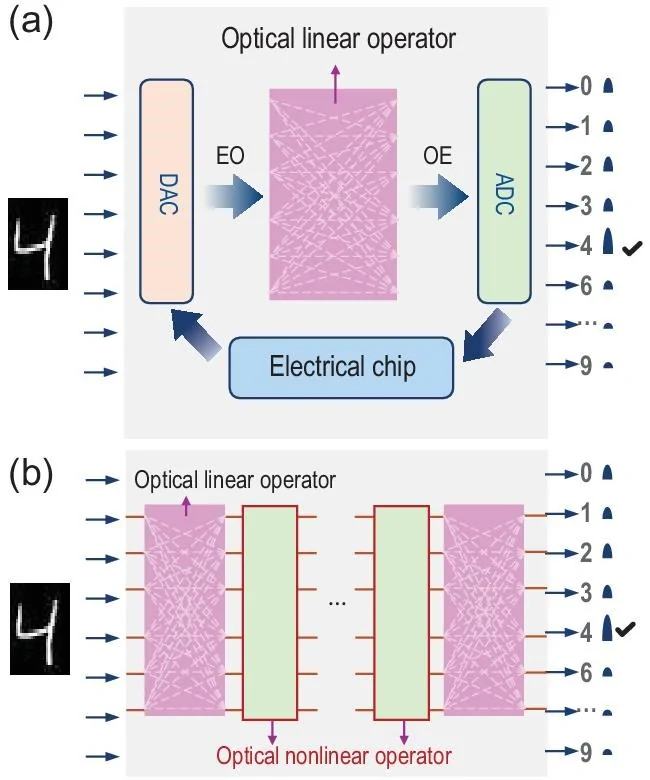
Two paradigms of optical computing. (a) Linear optical engines as coprocessors (communication between the optical operator and the electronic chip requires frequent optical–electrical and analog–digital conversions.). (b) Optical end-to-end computing (data remain fully in the optical domain). EO, electrical-to-optical; OE, optical-to-electrical; DAC, digital-to-analog converter; ADC, analog-to-digital converter.

However, this route is not a perfect solution for all problems. Deep neural networks are composed of alternating linear and nonlinear operations. Due to the lack of mature optical solutions for nonlinear activation functions, these hybrid systems require frequent signal conversions between optical and electrical domains, as well as between analog and digital formats, to implement nonlinear activation functions and deep networks. This recurrent conversion introduces significant latency and energy overheads, creating a bottleneck that can offset the gains from the optical MVM. The performance of the entire system becomes heavily constrained by the efficiency of these converters and the data movement between the optical and electronic components.

The second, more ambitious path aims to overcome this limitation by developing deep all-optical neural networks (ONNs). This approach involves integrating an optical nonlinear activation function directly into the photonic circuit, enabling end-to-end computation in which data remain in the optical domain from input to output ports, as shown in Fig. 1b. An ideal optical nonlinearity must satisfy several stringent requirements: it must be cascadable, meaning that it provides net gain to prevent signal decay over multiple layers; it must be compatible with large-scale silicon photonic manufacturing processes; it must have low energy consumption per activation; and it must operate at high bandwidth to match the speed of the optical MVM.

Achieving such a function has proven to be an enormous challenge. Attempts at optical nonlinearity on silicon platforms fall into two categories (all-optical and optoelectronic nonlinearity), each with critical trade-offs. All-optical nonlinearity relies on either integrating heterogeneous nonlinear materials with strong optical responses (e.g. III–V semiconductors, 2D materials) onto the silicon chip or the intrinsic nonlinearities in silicon or germanium. While the former is effective, heterogeneous integration complicates the fabrication process, increasing costs and potentially compromising reliability, thermal stability and scale. In detail [4], while thin-film lithium niobate offers foundry-compatible stability, its moderate Pockels coefficient necessitates millimeter-scale device lengths that fundamentally restrict the integration density. Conversely, electro-optic polymers leverage giant electro-optic coefficients to enable ultra-compact, energy-efficient footprints, yet their widespread adoption is impeded by high fabrication complexity and challenges regarding long-term thermal stability. Furthermore, although 2D materials offer the ultimate vertical scaling limit and zero-static-power activation, they currently face substantial fabrication barriers, particularly regarding uniform wafer-scale growth and damage-free transfer. While utilizing intrinsic nonlinearities in silicon or germanium ensures complementary-metal-oxide-semiconductor (CMOS) compatibility, the practical application is hindered by a trade-off: the thermo-optic effect is inherently slow due to thermal diffusion limits, whereas the Kerr effect suffers from low efficiency, necessitating high optical power densities [5]. These constraints ultimately impose a ceiling on both the bandwidth and the energy efficiency. On the other hand, optoelectronic nonlinearity offers a practical solution to achieve cascadable and CMOS-compatible ONNs [6,7]. However, its bandwidth is limited by the on-chip conversion process between optical and electrical domains. Consequently, a solution that simultaneously provides cascadability, compatibility and high bandwidth remains elusive, hindering the progress of truly deep ONNs.

The divergence of these two paths naturally leads to different application landscapes. The large-scale linear accelerator model is well suited for data centers and high-performance computing environments in which it can accelerate specific, well-defined tasks, particularly in deep-learning inference workloads, within a larger electronic workflow. In contrast, end-to-end ONNs, if realized, could unlock ultra-high-speed, low-latency applications at the edge, such as real-time image processing for autonomous vehicles or advanced radio-frequency signal processing, in which the elimination of the prohibitive optical–electrical and analog–digital conversion latency between optical hardware and CMOS circuits enables truly real-time computation.

Regardless of the structural distinctions between these two paths, a shared fundamental challenge lies in training these chips to achieve their desired functionalities. Non-reconfigurable devices typically rely on offline simulation-based training [8]; however, inherent fabrication errors frequently create discrepancies between simulation and physical reality, necessitating post-fabrication compensation. In contrast, reconfigurable architectures often employ *in situ* perturbation methods [9]. While this approach inherently accounts for imperfections, susceptibility to peripheral noise can compromise the gradient accuracy and convergence speed. Alternatively, surrogate modeling utilizes neural networks to approximate chip behavior for analytical gradient derivation. Yet, this method demands strict system stability and requires significantly larger datasets and complex architectures to accurately model high optical nonlinearity.

Looking forward, a critical challenge plaguing both approaches is static power consumption. In current silicon photonics, components such as MZIs and MRRs require continuous power for thermal tuning to maintain their programmed states, consuming significant energy even when the processor is idle. A promising solution lies in leveraging phase-change materials (PCMs) for in-memory optical computing [10]. PCMs, such as Ge₂Sb₂Te₅, can be switched between amorphous and crystalline states, exhibiting large nonvolatile changes in their refractive index. By integrating PCMs into photonic weights, it is possible to create nonvolatile optical memory elements that retain their programmed state without any static power draw. This would allow the optical processor to be powered down completely when not in use, drastically reducing the overall energy consumption. Nevertheless, to achieve practical, large-scale deployment, several critical challenges must be overcome: specifically, optimizing programming kinetics to facilitate rapid weight updates, ensuring robust endurance for prolonged operational lifespans and mitigating thermal crosstalk. Moreover, enhancing the multilevel storage capability is imperative, given its direct impact on computational accuracy. Beyond PCMs, the microelectromechanical system (MEMS) presents a viable alternative for low-power modulation [10]. While MEMS exhibits superior endurance compared with PCMs, it lacks nonvolatility; thus, it requires continuous refreshing (∼10 nW) to maintain its state and currently offers lower integration density than PCMs.

In conclusion, the field of optical computing is vibrant and rapidly evolving, but it must navigate a fundamental choice between immediate commercial viability and long-term disruptive potential. Building larger linear optical coprocessors offers a short-term solution for accelerating artificial intelligence workloads, despite the optical–electrical–optical conversion bottleneck. The quest for a deep end-to-end ONN, while far more challenging due to the lack of an ideal optical nonlinearity, represents the ultimate goal. To propel the entire field forward, the community must intensify its research on novel materials and devices for efficient nonlinear activation. Furthermore, embracing emerging technologies such as PCM-based in-memory computing to eliminate static power consumption will be crucial for unlocking the true promise of light-speed, ultra-low-energy computation.
